# Jennerian Medal

**Published:** 1801-05

**Authors:** T. Trotter

**Affiliations:** Plymouth Dock


*The following Address, with the* GOLD MEDAL, *voted by the Naval Medical Officers, has been presented to Dr. Jenner. Subjoined is a Liſt of these Gentlemen in Support of the New Inoculation.*

---

### To Dr. JENNER.

SIR,

YOU are, perhaps, no ſtranger to the information of the new Inoculation being directed throughout the Navy by Admiralty authority. The inquiries which had been inſtituted in the Channel for the laſt ſeven years, had called the attention of the ſurgeons to guard againſt the introduction of the Small-Pox

Pox among feamen; which in more than a hundred inftances during that time, had been imported by fhips; twenty of thefe have occurred within the laft fix months in this fleet only. Amidft fubjects fo ill prepared for its reception, more than the common proportion of deaths has been the confequence.—Such was the tenor of our refearches, when Dr. Jenner announced to the world the Vaccine Inoculation as a *prefervative* againft variolous infection: *Tandem venias augur Apollo.*

As far as the new practice has extended among us, it has been followed with the ufual fuccefs, and fo mild, that the fubjects of it have not been confidered in the number of fick on the lift. But the value of conducting the Vaccine Inoculation with fpirit and perfeverance throughout the navy, may be beft eftimated by calculating the feamen at 10,000, who are unconfcious of having had the Small Pox; in this proportion I am juftified by the experience of mufters in infected fhips. How dignified the councils of any nation, that by timely precaution fhall ward off fo much probable human mifery!

The medical officers have not been paffive fpectators of an event fo fingular in the hiftory of animated nature; an event which the Philofopher will contemplate with wonder, and the friend of his fpecies view with exultation. Although fecluded by their office from the earlieft communication with the progrefs of medical fcience, what relates to the Vaccine Difeafe has been earneftly fought after; and the whole of your opinions and practice have excited uncommon attention amongft us. I am therefore requefted to prefent you, in the name of thefe gentlemen, with a GOLD MEDAL, and fuitable devices; at once expreffive *of their fentiments in favour of the New Inoculation, and to commemorate its introduction into this department of public fervice*.* With the more pleafure I comply with the wifhes of my worthy affociates, as I am confident that no token of refpect beftowed on a benefactor of the human race, was ever conferred from more honourable or difinterefted motives. It will not be the lefs acceptable to Dr. Jenner, that it comes from a body of officers connected by the exercife of their profeffion with the moft brilliant period of our naval annals. As far as their authority has influence, they thus offer their warmeft fup-

* The medal reprefents Apollo as the God of Phyfic, introducing a young feaman recovered from the *Vaccine Inoculation* to Britannia, who, in return, extends a civic crown, on which is written, "JENNER." Above, "ALBA NAUTIS STELLA REFULSIT." Below, "1801."

On the reverfe, an anchor; over it, "GEORGIO TERTIO REGE;" and under it, "SPENCER DUCE;" expreffing the royal navy in the reign of George the Third, and naval adminiftration of Earl Spencer.

port to the cauſe. The progreſs of truth is ſometimes ſlow, but always certain. It is not in the nature of medical inveſtigation long to reſiſt the evidence of facts; and it is far leſs the province of medicine to check the current of charitable feelings, or to circumſcribe the duties of benevolence. We muſt therefore hope, that while the liberal diſcuſſion it has undergone ſhall ſecure the ſuffrages of the enlightened mind, the love of offſpring will confirm its favourable reception throughout domeſtic life.

Accept, Sir, in the name of my naval friends, my hearty congratulations on the honours that await your profeſſional exertions. May the preſent age have the juſtice and public ſpirit to remunerate what poſterity will be glad to appreciate; may the Medical Faculty have virtue and candour ſufficient to acknowledge the value of your labours; may your example be a model to the riſing members of that profeſſion which you adorn; and may you be bleſſed with length of days to ſee your diſcoveries the means of abridging and preventing diſeaſe, pain, and deformity throughout the habitable globe.

Incloſed, I have the honour to tranſmit a liſt of the medical gentlemen, and their ſtations in his Majeſty's naval ſervice. I beg, with all perſonal eſteem and regard, to ſubſcribe myſelf,

SIR, Your, &c.

T. TROTTER.

*Plymouth Dock, Feb.* 20, 1801.

---

### NAVAL SUBSCRIBERS to the JENNERIAN MEDAL, 1801.

Dr. THOMAS TROTTER, Phyſician of the Fleet.

| *Names.* | *Ships, &c.* | *Names.* | *Ships, &c.* |
|---|---|---|---|
| Wm. Hill, Surg. | Edgar | Dr. T. Kein | Royal George |
| James Veitch | Doris | B. M'Keirman | Prince |
| F. M. Chevers | Robuſt | B. M'Laughlan | Achille |
| W. Robertſon | Garland | H. Watſon | Cambridge |
| W. Hamilton | Clyde | W. Muſgrave | Triton |
| J. M'Intoſh | Galatea | J. Fraquhar | Captain |
| R. Allan | Oiſeau | C. Cooke | Penguin |
| A. Denmark | Viper | H. Hughes | Marlborough |
| T. Smith | Nimrod | T. Thong | Renard |
| J. Embling | Hoſp. Torbay | R. Cuming | Leyden |
| R. Hardwike | Magnificent | Dr. E. Boys | Hoſp. Deale |
| S. Hammick, jun. | Royal Hoſpital | W. M'Curdy | Pr. George |
| R. Lloyd | Barfleur | D. M. Bell | Indefatigable |
| An Admirer of Vaccinia | | Dr. C. Cudlip | Mill Priſon |
| | | G. Bellamy | Spencer |
| T. Stewart | Pr. Frederick | J. Milligan | Malta |

J. Bury

| *Names.* | *Ships, &c.* | *Names.* | *Ships, &c.* |
|---|---|---|---|
| J. Bury | Unicorn | S. Fowell | Spider |
| W. Gregory | Thames | W. M'Mullan | Canada |
| J. Landlefs | Hydra | T. Tofh | Triumph |
| T. Quin | Loire | T. Willes | Centaur |
| W. Gray | Nymphe | J. Young | Lion |
| J. Morgan | Amelia | T. Simpfon | Fifguard |
| M. M'Cormick | Immortalite | W. Beatty | Alcmene |
| W. M'Donald | Montague | A. French | Scout |
| Ral. Duke | Sprightly | P. Blair | Mars |
| Dr. J. Snipe | Hofp. Yarm. | H. Perkin | Urania |
| P. Cullen | Agamemnon | B. F. Outram | Superb |
| S. Allen | Albion | T. Mant | Terrible |
| W. Oaftler | Spitfire | J. Smithers | Royal Sov. |
| S. Hammick, fen. | Royal Hofpital | J. Allen | Formidable |
| G. M'Garth | Ruffel | T. Watherfton | Temeraire |
| R. Burke | Acteon | D. Fleming | Impeteux |
| W. Dingwall | Nemefis | R. Carruthers | Wareham |
| J. Leggat | Overyffel | J. Home | Suffifante |
| J. Scott | Audacious | G. Turnbull | Venerable |
| A. Leflie | Hazard | R. Thompfon | Childers |
| J. Boone | Solebay | Dr. M. Felix | Neptune |
| D. Cowan | Eurydice | D. M'Arthur | Naiad |
| D. S. M'Carthy | Beaulieu | R. Palin | Sylph |
| W. Nepecker | Jufte | M. Morgan | Mill Prifon |
| J. Nutt | Cæfar | F. Butt | Dragon |
| W. B. Smith | Pompee | R. Fairfoul | Cleopatra |
| R. Allen | Orion | Dr. Douglas Whyte, Conftantinople. | |
| A. L. Jack | Efpeigle | | |
| J. Lind | Majeftic | | |

